# Atypical Presentation and Evolution of Necrotizing Enterocolitis as a PIK3CA Pathological Variant

**DOI:** 10.7759/cureus.59243

**Published:** 2024-04-28

**Authors:** Nancy Laval, Niina Kleiber, Jean-François Soucy, Josée Dubois, Michael-Andrew Assaad

**Affiliations:** 1 Neonatology, Centre Hospitalier Universitaire Sainte-Justine, Montréal, CAN; 2 Neonatology, Centre Hospitalier Chrétien MontLégia, Liège, BEL; 3 Pediatrics, Centre Hospitalier Universitaire Sainte-Justine, Montréal, CAN; 4 Medical Genetics, Centre Hospitalier Universitaire Sainte-Justine, Montréal, CAN; 5 Radiology, Centre Hospitalier Universitaire Sainte-Justine, Montréal, CAN

**Keywords:** pros, overgrowth syndrome, late preterm, necrotizing enterocolitis, pik3ca

## Abstract

Activating mutation of PIK3CA is linked with cases of overgrowth syndromes and belongs to the PIK3CA-related overgrowth spectrum (PROS). Mutations in this gene are associated with vascular malformations, brain abnormalities, and an increased risk for certain tumors. We report the case of a newborn girl, preterm at 34 weeks of gestation, referred to our center for atypical necrotizing enterocolitis (NEC). At laparotomy, the appearance of the intestinal tract was described as puffy, cauliflower-like with a dark purplish coloration. Subsequently, the colostomy was described as having a consistent proliferative appearance. Medical treatment with sirolimus resulted in minimal improvement. There are no reported cases in the literature of association between NEC and PIK3CA mutation. It is possible that PIK3CA mutation, including the related vascular anomalies, plays a role in the pathogenesis of NEC with this condition.

## Introduction

The phosphatidylinositol 3-kinases (PI3K) are a group of lipid kinases discovered in the 1980’s. PI3K and especially its catalytic subunit alpha (PIK3CA) regulate the signalizing pathway involved in cell proliferation, migration, and survival [1]. Activating mutations are identified in tumorigenesis, as reported by Samuels st al. in 2004 for the first time in human breast cancer [2].

The phenotype associated with PIK3CA-related overgrowth syndrome (PROS) can vary in severity, ranging from mild conditions like isolated macrodactyly to severe multi-organ involvement. The variability in expression is closely linked to the timing (whether the mutation is germline or somatic) and the specific location of the mutation within the body [1]. Germline mutations in PIK3CA typically arise during embryonic development and are associated with more widespread and severe overgrowth that affects multiple organs [3,4]. In contrast, somatic mutations result in localized overgrowth in specific tissues or regions.

Keppler-Noreuil et al. conducted a comprehensive review of various clinical entities related to PIK3CA mutations and defined diagnostic criteria in 2015 [5,6]. In newborns, common clinical features include macrosomia, asymmetric musculoskeletal development, and vascular and lymphatic malformations [7,8]. Brain anomalies such as capillary malformation, isolated polymicrogyria, or dysplastic megalencephaly have also been reported [9,10]. In some rare cases, individuals may present with primary tumors, such as nephroblastomatosis [11]. Germline mutations have been identified in cases characterized by macrocephaly, megalencephaly, or limb overgrowth [12,13].

In this report, we present a unique clinical presentation of a PIK3CA mutation associated with necrotizing enterocolitis (NEC), which has not been previously reported. NEC is an acute inflammatory disease of the bowel that requires prompt action and remains an important cause of morbidity and mortality in newborns. Although incidence decreases with gestational age and higher birth weight, it occurs between 5% and 25% of term and late preterm babies [14,15]. Common risk factors include prematurity, small for gestational age (SGA), congenital heart disease (CHD), hypoxic-ischemic encephalopathy, and uncommonly polycythemia, thrombophilia or sepsis [15,16].

We hypothesize that microvascular anomalies found in association with the PIK3CA mutation may have led to reduced mesenteric perfusion and thus contributed to the development of NEC.

## Case presentation

A preterm neonate was transferred to our neonatal intensive care unit (NICU) for management of atypical NEC. Labor was induced at a referral hospital at 34 weeks 0/7 due to suspected rupture of membranes (ROM) at 31 weeks, pre-eclampsia, and fetal macrosomia. Antenatal corticosteroids were received at 31 weeks due to suspected ROM. This was a third pregnancy, the two previous ones being spontaneous abortions. Third-trimester ultrasound revealed polyhydramnios and macrosomia without gestational or pregestational maternal diabetes. No malformations were detected. There was no report of consumption of toxins or drugs during the pregnancy and serologies were protective.

**Figure 1:**
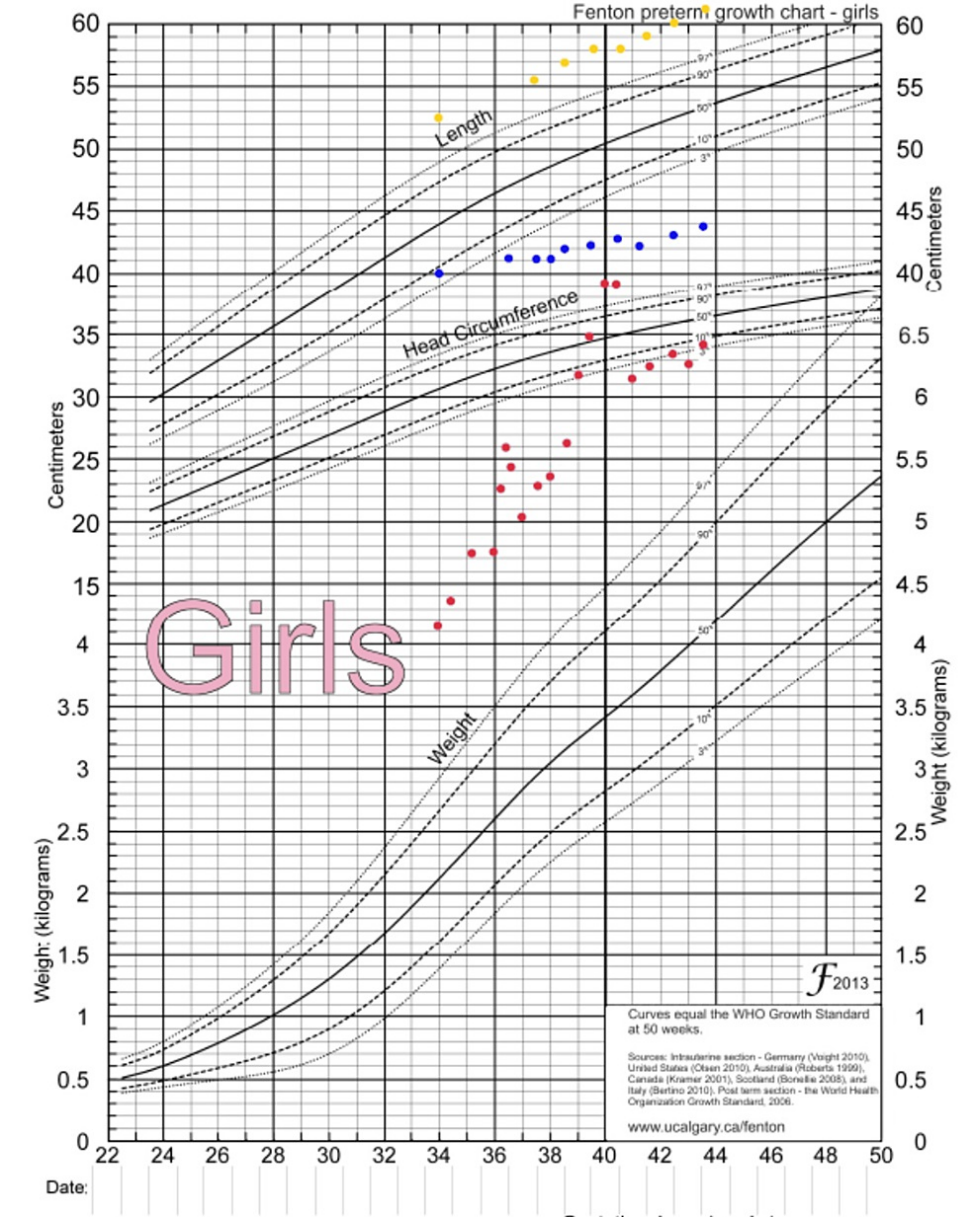
Growth chart Patient’s growth parameters demonstrating significant overgrowth: weight (red dots), head circumference (blue dots), and length (yellow dots). Measures plotted on the Fenton growth chart for preterm infants [17]; (used with permission, available at www.biomedcentral.com.)

Delivery was by cesarean section due to abnormal fetal tracing (fetal tachycardia). The baby cried at birth but had a secondary apnea requiring positive pressure ventilation. Apgar scores were 1/3/7. There were no signs of birth asphyxia (arterial cord pH 7.30). Birth measurements were: weight 4,160 g (+4.48 SD); height 52.5 cm (+3.21 SD); cranial perimeter 40 cm (+6.29 SD) (Figure 1).

A brief episode of atrial flutter was noted in the first hours of life, converting spontaneously to sinus rhythm, for which she was treated with oral sotalol which was stopped on the day of life (DOL) nine. The baby required continuous positive airway pressure (CPAP)(PEEP 7) with a FiO2 of 30% for 24 hours. An umbilical venous catheter (UVC) was inserted at birth for transitory hypoglycemia. Feeds were started at DOL2 and comprised of breast milk supplemented with formula. At DOL4, CPAP was restarted for respiratory distress and abdominal distension. Abdominal radiography revealed pneumatosis and standard treatment for NEC (nil per os, blood culture, large bore nasogastric suction catheter total parenteral nutrition (TPN), and broad-spectrum antibiotics: piperacillin-tazobactam) were instituted at the referral center. Blood gas, complete blood count (CBC), and c-reactive protein (CRP) at this time revealed normal values. At DOL6, platelets and white blood cell count (WBC) also dropped to 68 x 10^9/L and 3.2 x 10^9/L respectively and CRP increased to 64 mg/L. At DOL7, she required her first platelet transfusion (42 x 10*9/L). A peripherally inserted central catheter (PICC) was installed at DOL8 with the removal of the UVC. Platelets remained stable between 50-100 between DOL8-10, but on DOL11 she required a second platelet transfusion, prompting a call to the tertiary care center. No metabolic acidosis during her stay at the referral center. 

**Figure 2:**
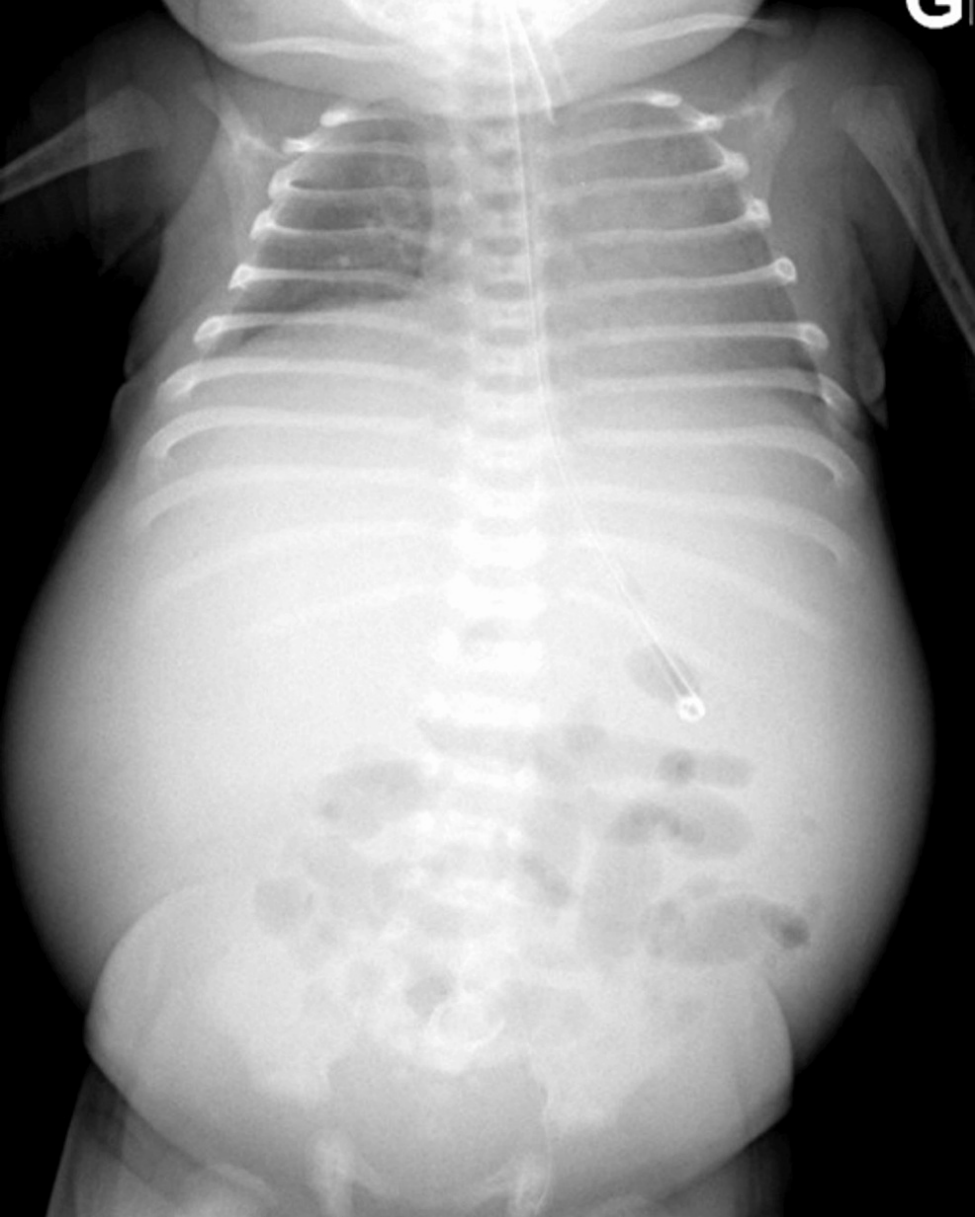
Supine abdominal x-ray at day 11

The infant was transferred to our unit at DOL11 due lack of clinical improvement despite optimal treatment for NEC. Abdominal distension, redness, and hematochezia were noted upon admission. Blood test results revealed inflammation, with a CRP level of 139mg/L and thrombocytopenia at 116 x 10^9/L and severe respiratory acidosis (pH 7.04, pCO2 93 HCO3 24), prompting rapid intubation. The coagulation profile revealed an INR of 1.62, activated partial thromboplastin time of 41.4 seconds, and a fibrinogen level of 2.92 g/L. Abdominal radiography and ultrasound revealed a significant amount of free fluid in the abdominal cavity without signs of pneumatosis, portal venous gas, or perforation (Figure 2). 

**Figure 3:**
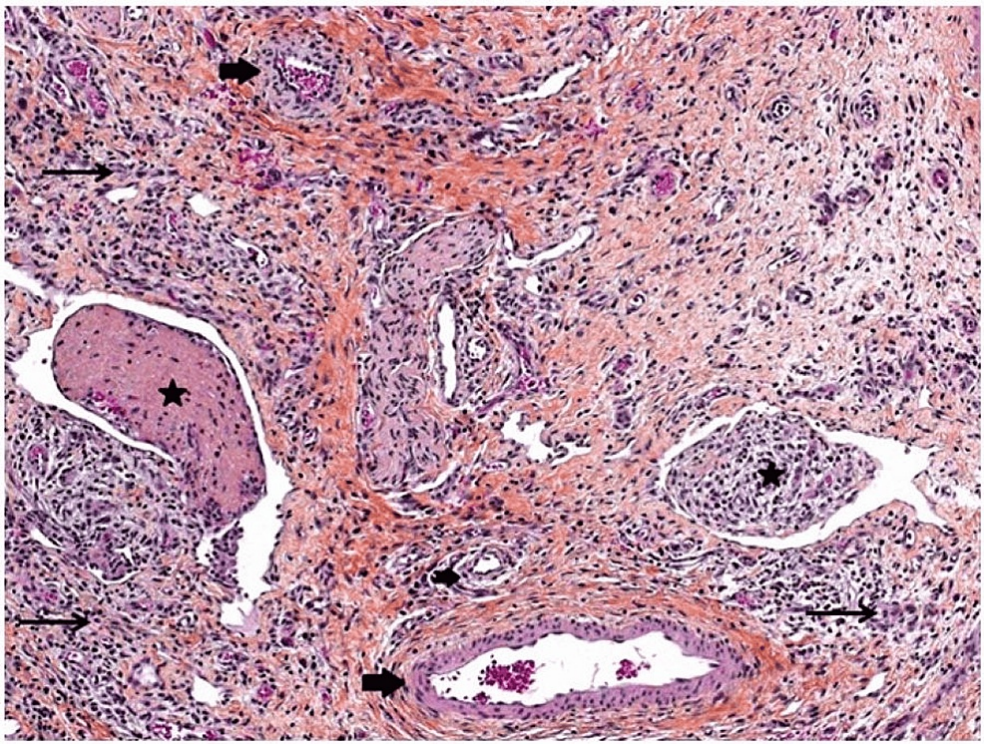
Hematoxylin-eosin stain pathology image of transverse colon Abnormal presence of vessels in the subserosal layer with multiple thrombi with abundant arterioles (big arrows) and venules (stars) containing organized thrombi. The thin arrows show the importance of granulation tissue with neutrophilic infiltration, a sign of peritonitis.

A diagnosis of atypical NEC was made due to the absence of classic radiologic signs of NEC and the patient's gestational age without any other obvious comorbidities increasing the risk of NEC (cardiac disease or low perfusion state). On DOL13, due to increasing abdominal distension and respiratory decompensation (switched to high-frequency oscillatory ventilation), an exploratory laparotomy was performed. The surgery revealed diffuse inflammation of the terminal ileum without any evidence of perforation. The intestines had an unusually dark appearance and were notably swollen. Pathological examination of the intestines revealed ischemic necrosis and an unusual abundance of blood vessels in the subserosal layer, along with multiple thrombosed vessels (Figure 3).

**Figure 4:**
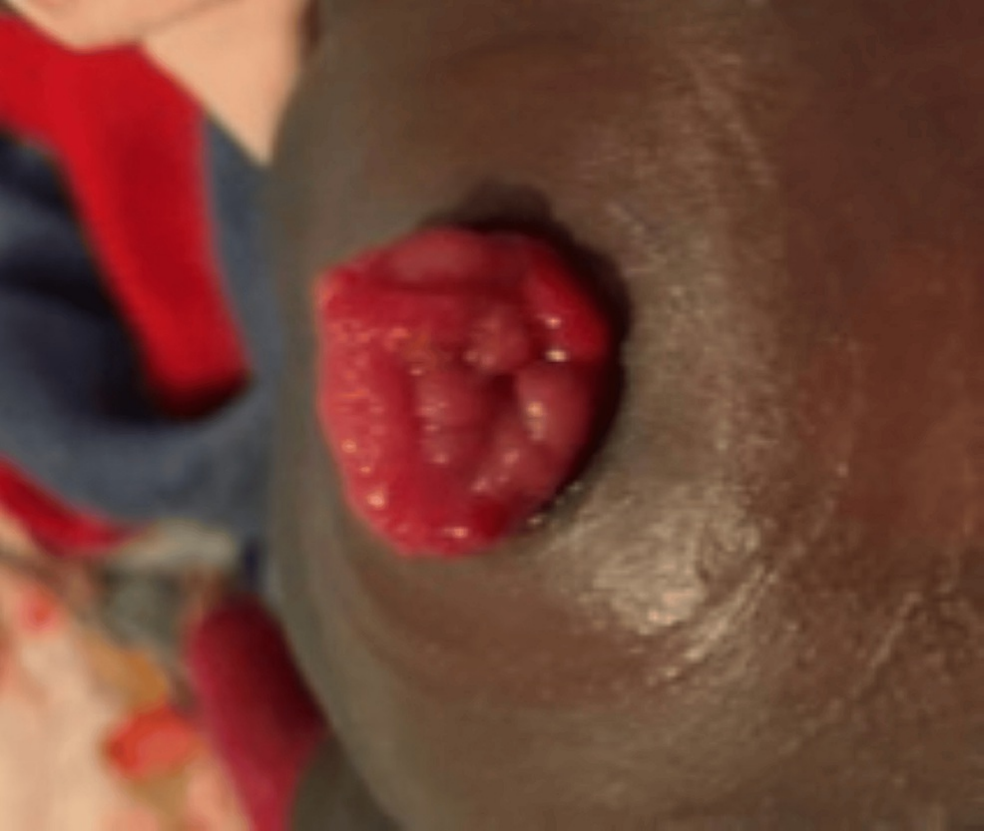
Colostomy Aspect 4 weeks after surgery. Note the atypical cauliflower-like appearance, indicative of overproliferation.

The patient was extubated to CPAP six days after her first surgery and later to room air. Feeds were restarted on postoperative day eight and progressed slowly up to 90 ml/kg/day. On DOL32, the patient developed central-line sepsis with shock, abdominal distension, and respiratory distress requiring re-intubation, fluids, and antibiotics. Due to severe abdominal distension, computed tomography of the abdomen was performed at DOL33, revealing likely colonic stenosis with a significant amount of ascites. On DOL35, she went to the operating room for a second time revealing intestinal obstruction due to stenosis of the left colon and significant adherences. She required resection of her descending colon and a colostomy in her transverse colon. The intestines were once again found to have an unusually dark appearance. Her second post-operative course was challenging with significant inflammation requiring multiple fluid boluses and vasopressors. She was successfully extubated 11 days after her colectomy and feeds restarted at 14 days post colectomy. Although the clinical course in the NICU was generally favorable, the stoma displayed a cauliflower-like shape and appeared to be actively proliferating (Figure 4).

A 180k array comparative genomic hybridization test yielded normal results. However, a next-generation sequencing panel for overgrowth syndromes on peripheral blood identified a known pathogenic variant in the PIK3CA gene (NM_006218.3:c.2176G>A, p.Glu726Lys), present at a 24% variant allele frequency in the tested specimen.

Extra-intestinal findings

**Figure 5:**
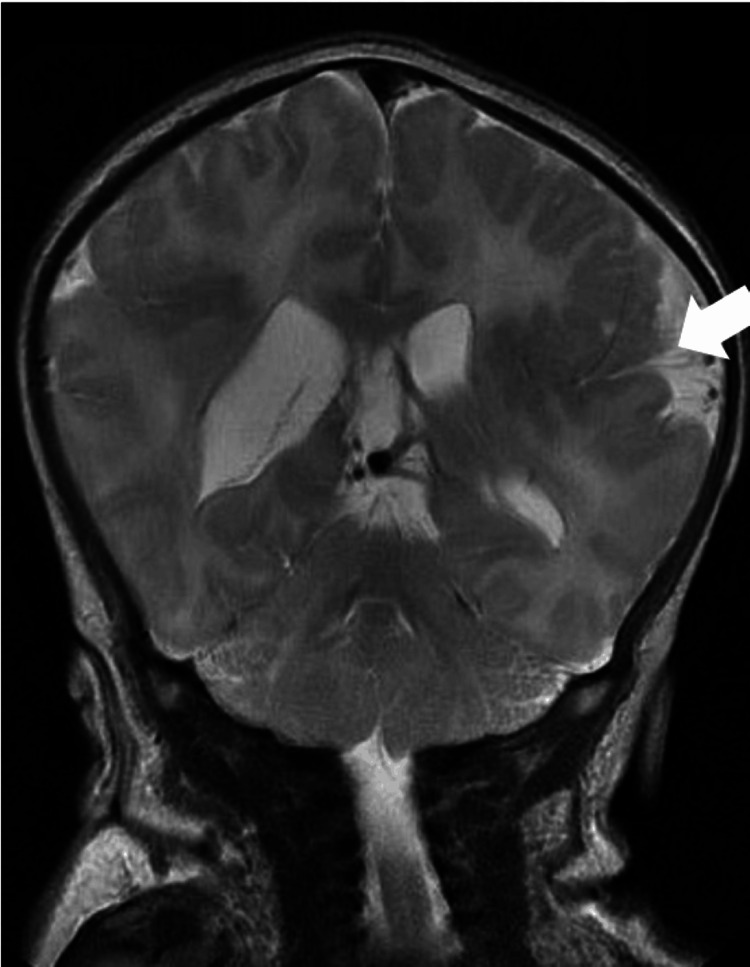
Brain MRI Note polymicrogyria at the frontotemporal junction bilaterally with extension to the insula on the right (white arrow).

All the baby’s growth parameters were above the 99th percentile (Figure 1). After her first surgery, increased attention was given to her neurological examination which was found to be very abnormal with hypotonia and poor visual contact. Magnetic resonance imaging (MRI) was performed on DOL27 of the brain and showed polymicrogyria at the frontotemporal junction bilaterally with extension to the insula on the right without massive hydrocephalus or Chiari malformation (Figure 5).

**Figure 6:**
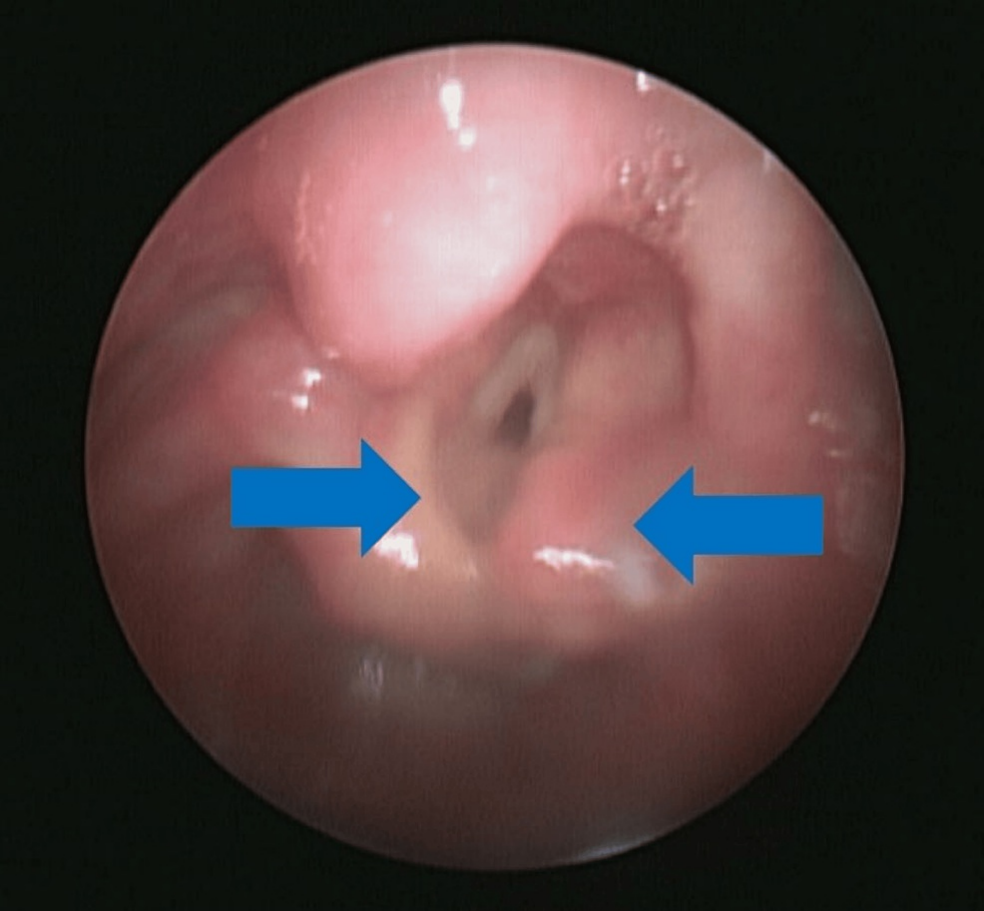
Nasal endoscopy Significant tissue proliferation around arytenoid cartilage (blue arrows).

There was also significant overgrowth of arytenoids resulting in recurrent obstructive apneas (shown in Figure 6), requiring chronic positive pressure ventilation while in the NICU, as well as two supraglottic surgeries to resect tissue obstructing the airway. Multiple abdominal ultrasounds were performed during her NICU stay, revealing normal kidney architecture. She experienced mild renal failure during her episode of septic shock. 

As previously described, the patient had atrial flutter at DOL2, resolving spontaneously but treated with sotalol until DOL9. Her echocardiogram was otherwise normal. The baby also suffered from several venous thromboses at sites of central venous catheters, notably in the left portal vein, as well as in deep lower limb veins, for which treatment with enoxaparin was instituted. On outpatient follow-up, it was noted that some of these thrombi continued to progress despite anticoagulation, which she required until her death. The patient was started on sirolimus on DOL83 with some clinical improvement (removal of ventilatory support, ability to feed with bottles) and was discharged from the NICU on DOL91. The mammalian target of rapamycin (mTOR) integrates signals from the phosphatidylinositol 3-kinase (PIK3)/protein kinase B (AKT) pathway to coordinate cell proliferation and survival [18]. The PIK3/AKT/mTOR pathway is essential for normal blood vessel development and the overactivation of this pathway seen with PIK3CA mutations is associated with vessel and tissue overgrowth [19]. Sirolimus inhibits mTOR signaling, which inhibits cell proliferation [18]. It is widely used off-label for the treatment of vascular anomalies and associated overgrowth [20]. 

In the following months, she developed progressive macrocephaly, for which neuroimaging was reperformed at six months of age, revealing hydrocephalus due to cerebella tonsillar ectopia (Chiari malformation). She was referred to neurosurgery and had a ventriculoperitoneal shunt inserted at seven months of age. She passed away at nine months of age at home from a cardiorespiratory arrest of unknown origin.

## Discussion

The PIK3CA c.2176G>A variant is a known pathogenic missense variant, as reported in ClinVar by the ClinGen Brain Malformations Variant Curation Expert Panel [21]. It is absent from the gnomAD dataset [22]. It causes glutamine to lysine substitution at position 726, which is highly conserved. International recommendations suggest performing genetic analysis on affected tissue [4]. In our patient, the mutation was found with a variant allele frequency of 24% in peripheral blood, likely explained by a post-zygotic acquisition of the mutation at an early embryonic stage. It may also explain the severity of the clinical manifestations. Megalencephaly-capillary malformation-polymicrogyria syndrome (MCAP) is a clinical form of PROS [23]. In a recent study of 12 patients, 92% had macrocephaly and 83% had Arnold-Chiari malformation type 1 [9]. Other clinical abnormalities were found such as cutaneous vascular malformations and digital anomalies. Our patient presented with many features of MCAP, but had additional findings not previously described. 

NEC

To the best of our knowledge, there are no documented cases of NEC occurring in a preterm infant with a PIK3CA mutation in existing literature. Vascular anomalies associated with PROS can manifest in various forms, including venous, capillary, arterial, or a combination thereof [24]. These anomalies encompass malformations of vascular walls and excessive vessel proliferation, along with an elevated susceptibility to venous thrombosis [1,24]. However, even in the absence of vascular malformations, there is a heightened risk for thrombosis which can be attributed to vascular endothelial dysfunction and particular endothelial adhesion molecules found in individuals with PIK3CA mutation [1,7,24]. Histopathological analysis of this case showed an aberrant proliferation of blood vessels in the subserosal layer, along with multiple microthrombi (Figure 3), aligning with observations from the existing literature.

It is conceivable that an interplay between excessive cellular growth, insufficient vascularization with resultant ischemia, and microthrombi contributed to the diagnosis of NEC. Clinicians should remain attentive to the possibility of mesenteric hypoperfusion in individuals with overgrowth syndromes, particularly those with known vascular involvement.

Other clinical findings

In individuals with a PICK3CA-related disorder, the presence of polymicrogyria, as observed in our patient, heightens the likelihood of encountering developmental delays, intellectual disabilities, epilepsy, and muscle tone irregularities [7]. While cases of apnea have been reported in such patients, they primarily stem from central origins [1]. In contrast, our patient presented with obstructive apnea attributed to laryngeal tissue proliferation.

## Conclusions

In summary, this represents the first documented instance of a neonate exhibiting an atypical case of NEC with a PIK3CA overgrowth mutation. This case underscores the significance of acknowledging the multisystemic impact stemming from the remarkably early acquisition of this mutation and its high allelic prevalence. Clinicians should be vigilant about the potential risk of mesenteric hypoperfusion related to vascular involvement in overgrowth syndromes, particularly in cases of premature birth.
